# Assessing Activity and Location of Individual Laying Hens in Large Groups Using Modern Technology

**DOI:** 10.3390/ani6020010

**Published:** 2016-02-02

**Authors:** Janice M. Siegford, John Berezowski, Subir K. Biswas, Courtney L. Daigle, Sabine G. Gebhardt-Henrich, Carlos E. Hernandez, Stefan Thurner, Michael J. Toscano

**Affiliations:** 1 Animal Behavior and Welfare Group, Department of Animal Science, Michigan State University, 474 S. Shaw Ln, East Lansing, MI 48824, USA; siegford@msu.edu; 2 Veterinary Public Health Institute, Vetsuisse Fakultät, University of Bern, Schwarzenburgstrasse 155, Liebefeld CH-3097, Switzerland; john.berezowski@vetsuisse.unibe.ch; 3 Department of Electrical and Computer Engineering, Michigan State University, 428 S. Shaw Ln, East Lansing, MI 48824, USA; sbiswas@msu.edu; 4 Department of Animal Science, Texas A & M University, Room 133 Kleberg, 2471 TAMU, College Station, TX 77843, USA; cerldaigle@gmail.com; 5 Center for Proper Housing: Poultry and Rabbits (ZTHZ), Division of Animal Welfare, VPH Institute, University of Bern, Burgerweg 22, Zollikofen CH-3052, Switzerland; sabine.gebhardt@vetsuisse.unibe.ch; 6 Department of Animal Nutrition and Management, Swedish University of Agricultural Sciences, P.O. Box 7024, Uppsala SE-750 07, Sweden; carlos.hernandez@slu.se; 7 Bavarian State Research Centre for Agriculture, Institute for Agricultural Engineering and Animal Husbandry, Voettingerstrasse 36, Freising 85354, Germany; Stefan.Thurner@LfL.Bayern.de

**Keywords:** tracking, individual, activity, RFID, motion, production

## Abstract

**Simple Summary:**

Tracking of individual animals within large groups is increasingly possible offering an exciting opportunity to researchers. Whereas previously only relatively indistinguishable groups of individual animals could be observed and combined into pen level data, we can now focus on individual actors and track their activities across time and space with minimal intervention and disturbance. We describe several tracking systems that are currently in use for laying hens and review each, highlighting their strengths and weaknesses, as well as environments or conditions for which they may be most suited, and relevant issues to fit the best technology for the intended purpose.

**Abstract:**

Tracking individual animals within large groups is increasingly possible, offering an exciting opportunity to researchers. Whereas previously only relatively indistinguishable groups of individual animals could be observed and combined into pen level data, we can now focus on individual actors within these large groups and track their activities across time and space with minimal intervention and disturbance. The development is particularly relevant to the poultry industry as, due to a shift away from battery cages, flock sizes are increasingly becoming larger and environments more complex. Many efforts have been made to track individual bird behavior and activity in large groups using a variety of methodologies with variable success. Of the technologies in use, each has associated benefits and detriments, which can make the approach more or less suitable for certain environments and experiments. Within this article, we have divided several tracking systems that are currently available into two major categories (radio frequency identification and radio signal strength) and review the strengths and weaknesses of each, as well as environments or conditions for which they may be most suitable. We also describe related topics including types of analysis for the data and concerns with selecting focal birds.

## 1. Introduction

Scientific and technological advances are being made at an ever-increasing rate, changes which deliver continuous benefits to Animal Welfare science and the associated practicalities of assessment. The research community must take full advantage of these advances to generate more relevant and accurate portrayals of animal experiences and understanding of how they interact with the surrounding environment. The variation of animal behavior and needs at the individual animal level is a well-known phenomenon within our discipline. Individual animals within pens can have unique effects upon their penmates and thus the group or pen needs to be considered as an experimental unit for appropriate statistical analysis [1]. However, welfare is an individual experience, as affective states such as pain, suffering, and hunger can only be experienced at the individual level [2,3]. An example of a similar issue with related consequences to animal welfare is that commercial diets are produced for the “average” animal within a group, with possible adverse consequences at the extremes of nutritional requirements, e.g., some animals will get too much of a nutrient for their individual specific needs while others will get too little [4]. Nonetheless, advances in technology have proved innovative in solving the problem of meeting individual nutritional needs (at least for larger animals) in the form of electronic tags recognized by a feeding system that allows the provision of a diet tailored for that individual animal [5] and consequent likely improvements to animal welfare. While it could be argued as to whether the system is cost-effective or allows all nutritional needs to be met, the point is that technology—specifically development of miniature tracking systems integrated into larger husbandry management systems (*i.e.*, feed delivery devices)—provides a solution to meet individual animal needs. This change will likely improve animal welfare at the individual level in comparison to generically feeding the “average” animal.

When charged with the objective of improving the welfare of animals, the capacity to form assessments at both the individual and group levels can alter the accuracy of how we judge the welfare of animals. Given the global trend for increasing size of commercial animal production units and the reduced time stockmen spend with animals in their care, assessments and research at the group level are common. The pattern places us at a critical juncture where the development of technology could (and likely should) allow for a paradigm shift in how we assess animal welfare. Individual hens, previously lost amongst a crowd of conspecifics and group averages, can now be tracked, assessed, and evaluated to ensure their welfare meets criteria that are relevant. Such technology also allows for a richer understanding of individual variability, adaptability, and coping in current and proposed production systems. Our research community, with the common goal of improving (and possibly ensuring) the welfare of animals cared for and kept by humans, has the moral obligation to adapt to these new conditions and use all the tools of science available for this charge. We must make changes in how Animal Welfare science is conducted or risk our experimental conclusions being irrelevant to the actual welfare of individual animals.

More specifically, the problems facing the contemporary laying hen industry from a welfare perspective are considerable and would greatly benefit from the ability to track individual animals. The technology is particularly relevant given the global movement from battery caged housing systems characterized by less than 10 hens per cage to alternatives. Common alternatives are extensive systems including aviaries, barns, and free-range that may house hens in groups of thousands. While movement away from battery cages is believed to be associated with a variety of benefits to animal welfare including increased performance of natural behaviors [6], alternatives to battery cage systems also introduce a host of unique problems [7]. For instance, damage to the keel bone is believed to increase in extensive systems as a result of collision with internal housing structures despite the increased bone strength that should result from the greater range of movement [8]. Of particular relevance for this manuscript, extensive systems represent a remarkable research challenge because of the increased size of the flock. Extensive systems typically house hens in groups upwards of 15,000 nearly identical birds, making observation of individual animals by traditional methods impossible. With the dramatic change in housing type and the development of large flocks, methodologies that allow identification, tracking, and study of individual hens in these large groups and complex environments are essential.

In an effort to describe how advances in tracking technology can improve the welfare of poultry, the present paper is divided into several sections to assist researchers to design and prepare for experiments that seek to track individual bird activities and location within large groups. Given the unique and challenging conditions of large-scale poultry systems, this information will be of particular use to investigators of poultry welfare. We initially discuss tangential issues that relate to this technology and its deployment and then provide an overview of how technology can be used to remotely identify behaviors being performed as well as generate information regarding production and location for individual animals. Within each system, we review several instances in applied settings to showcase the practical aspects of each and how limitations can be circumvented. A summary table is provided of the various methods reviewed (Table 1). 

**Table 1:** Summary of strengths, weaknesses, primary and previous applications of radio frequency identification and radio signal strength systems used in laying hens.

	Radio Frequency Identification	Radio Signal Strength
Primary application differences	Detects presence or absence of individual at location of antennae	Detects movement of individual through time and space and possibly location
Strengths	Possibility to couple with other systems Can detect multiple individuals simultaneously No external power source or battery needed (for bird-mounted component) Relatively easy installation and mobile Sensor can be placed at several points on the body	Ability to couple with various other observations including: accelerometry (described in this article), light, temperature, humidity
Weaknesses	Water or metal can dampen signal strength Quickly moving (or immobile) animals difficult to detect Requires multiple antennae to detect direction of movement Expensive, though depends on system Mainly in high frequency systems (>13.56 MHz), conspecifics can block signal causing missed readings	Stationary receivers ≥1 meter apart Overlapping detection fields Short battery life Metal can obstruct detection causing data loss Sensor must be placed on back of hen for best signal capture
Previous applications	Passage through a pop hole Egg laying in a nest box	Location relative to other sensors (both stationary and mobile) Individual behavior and movement in large groups, including within 3 dimensions

## 2. Statistical Analysis for Individuals within Large Groups

In poultry research, studies that measure productivity or performance and associate risk factors for their improvement are most often based on data collected at the group (e.g., pen or flock) level. These studies are both convenient and useful, because the unit of production in the poultry industry is almost exclusively the group rather than the individual. However, studies with a group-level focus have limitations when interpretations are needed at the individual bird level, particularly when animal welfare is a concern. 

The most well-known limitation for associating group-level data to risk factors for the individual is referred to as ecological bias or fallacy [9]. Ecological fallacies are invalid (false) inferences that are made about individuals when outcomes, or risk factors for those outcomes, are measured at the group level and not at the individual level. With group level measurement, it is not possible to know whether the individuals in the group that experience the outcome of interest are the same individuals in the group that experience the risk factor. If they are not the same individuals, then the inferences made about associations between the outcome and risk factor may be biased. In behavioral studies, ecological bias may result when birds in a flock do not perform a behavior to the same degree (some not at all and some to a very high degree). For example, individual feeding behavior, or the amount of time that each bird spends feeding or the amount of feed that each bird consumes at a feeding bout, is likely to vary considerably between individual birds. These individual bird behaviors could potentially be risk factors for outcomes such as egg laying. If a risk factor is measured only at the flock level (for example, daily feed consumption for the flock) and the outcome is also measured at the flock level (total eggs produced for the flock per day), it will not be possible to determine if birds that consume more feed actually produce more eggs (or *vice versa*). In regards to behavior, hens living in groups experience social pressures from other hens, and if a hen is perceived as having low social status, she may be chased, pecked or attacked when she moves around the environment and attempts to feed, drink, nest or perch. Thus social risk factors can vary greatly between individual birds and are of considerable importance to hen welfare, as feather pecking, cannibalism and injury due to aggression are prevalent issues. Studies that do not measure risk factors (e.g., feeding or social behavior) and outcomes (e.g., egg laying or injury) for each individual bird will be at risk of having biased results. Other studies such as those estimating the effect of environmental conditions (e.g., room temperature, humidity, and light) are unlikely to be affected by ecological bias because all birds experience the same level of exposure to these conditions. For instance, if the light exposure of each hen in the flock were measured, the differences would be negligible. Alternatively, given the complex environments of housing systems like aviaries, there are likely to be micro-climates that individual hens can enter and exit (in comparison to battery cages which eliminate movement to new locations) requiring that even these factors be considered. 

Additionally, ecological bias can apply to both cross sectional studies (at one point in time) and longitudinal studies (repeated measurements or time series data). Application of technologies that allow measurement and monitoring of individual bird characteristics that vary over time provides a tremendous opportunity to gain a new understanding of bird behavior, productivity, disease, and other factors of interest. Once collected, these time series data can be analyzed in a purely descriptive manner (describing characteristics of interest) or they can be used to test hypotheses about the associations between various behaviors or other characteristics of interest.

The time interval during which individual bird characteristics are measured or aggregated will influence the statistical methods that are appropriate and also the inferences that can be made from the measurements. In some studies, time periods may be over a wider interval; for example measurements made each week, month or once in a production cycle where diurnal or seasonal rhythms may manifest. In these longitudinal studies, time is often considered a discrete, repeatedly measured variable and analysis is often with repeated measures ANOVAs or generalized linear models [10,11]. In other studies individual measurements may be recorded in real-time (continuously) or in near-real-time and are analyzed using time series analysis methods that model continuous changes in individual birds or groups of birds over time. A review of statistical methods for time series analysis is provided by Chatfield *et al*. [10]. Comparing groups of time series analysis can be done with generalized linear models that can include variables measured at both individual bird and group levels (e.g., modeling individual bird behaviors such as eating, drinking, and movements with group level measurements such as daylight cycle, or barn temperature). Also of interest may be multivariate models that assess the association between predictor variables and multiple outcome variables, e.g., measuring the association that several potential risk factors have with both individual egg production and health status. Another approach would be to develop simulation models that can be used to infer other properties about the population under study or that could be used to test the effect of various scenarios or group level factors on individual birds over various time periods. Individual bird measurement data could be used to parameterize agent based models [12] (simulation models in which individual agents are given properties and allowed to interact over time within the model space). These models could for instance be used to estimate outcomes (such as egg production) that may result from simulated populations made up of different mixtures of birds each having different susceptibility to leg injury and or feeding behaviors. 

At the analytical level, identifying statistical associations requires the comparisons of groups of hens with different risk factors or outcomes of interest. For example, we might compare individual outcomes based on the amount of time a bird spends roosting. In this comparison, we could identify birds that individually spend more time roosting and compare this group to a second group of birds that individually spend less time roosting, all within the same physical environment (e.g., pen). 

Lastly, it is critical in group-level studies to ensure that the study sample is representative of the flock to which the researcher plans to generalize the study findings. Failing to do this can result in the introduction of selection bias—bias that results from inappropriate selection of study subjects—into the study. More simply, if the individual birds in the study sample are different from those in the population, then the study results are likely to be differ from the population causing inappropriate conclusions to be drawn [13]. Approaches for mitigating selection bias are discussed in Section 4. 

It is important to point out that individual and continuous measurement of birds over time will not guarantee elimination of selection bias. A small sample of birds selected from a large flock may still be a biased sample, even if birds are individually measured and monitored. It is only when randomly selected birds that are representative of the population or very large numbers of birds are monitored over time that selection bias can be reduced or eliminated. With very large samples it is possible to group individuals in the sample into smaller and more uniform sub-groups based on one or more characteristics of interest. If the sub-groups are indeed relatively uniform then associations identified for that sub-group may be generalized to other populations, but only to subgroups with similar characteristics. Continuous collection and analysis of individual data from large numbers of birds, similar to the numbers seen in large commercial flocks, is currently a technological and analytical challenge. Individualized monitoring in human health (for example mobile health technology) is gaining popularity and may provide some analytical methods that are transferrable to animal studies [14]. However, there are considerable differences between the types of data collected for poultry and humans and few of these methods have been applied in poultry health and welfare. In addition, the current costs of continuous individual monitoring devises prohibit their large-scale application in poultry production (see Section 4). 

## 3. Inference between Hen Health and Activity

It is generally accepted that a sick animal has poor welfare [15]. Animals behave differently when they are ill, injured, or distressed and changes in behavioral choices can be reflective of changes in the individual hen’s health or social status. Sick animals show quantifiable decreases in feeding, drinking, activity, and social contact as well as increases in resting, huddling, and shivering [16,17]. Further, as hens age, they become more variable in their behavioral choices with changes in home range size and location as well as in their proximity to other individual hens [18]. Therefore, changes in behavioral patterns can be used as a proxy measure for how the animal may be feeling and its overall welfare state. However, in order to treat an animal in poor health, it must first be identified as ill, a task that is becoming increasingly difficult with ongoing trends in poultry production towards larger and more populated group units such as aviaries and enriched cages. 

While these recent developments in housing systems (e.g., use of furnished cages or aviaries) are likely to improve the welfare of animals overall through greater ability to perform natural behaviors that are driven by internal motivations [6], they also introduce novel problems as well. The large size of groups in furnished cages or aviaries can make it difficult for caretakers to visually assess the health and welfare of individual hens or to monitor changes in individual hens over time, particularly when characterized by a relatively homogenous appearance. Compounding this problem is the natural tendency of animals to suppress signs of sickness in order to avoid aggression from other members of the group or attention from predators [17]. Relative to laying hens housed in small, simple conventional cages, sick hens in non-cage or large, enriched cage systems have a greater ability to hide among other hens or equipment in the system, slowing detection of sick hens. Further, many of the initial behavioral changes associated with sickness are subtle (e.g., decreased activity or feeding) or are designed to remove the animal from notice (e.g., hiding or seeking isolation) [17]. Some changes may also occur transiently during the acute phase of an illness. Thus, with the exception of the grossest of injuries, behavioral changes associated with sickness are currently nearly impossible to detect in one laying hen housed in a group of many identical looking hens. 

Given these concerns, a component of successful adoption of housing systems for poultry that allow expression of natural behaviors but do not compromise welfare by increasing sickness will be the ability to monitor animals. Effective monitoring will be required in order to detect disease problems in individual hens quickly enough to alleviate the problem in that hen or prevent it from affecting an entire flock. The use of technology that can intelligently monitor animal behavior (e.g., specific activities such as feeding, walking, or remaining still) and location, coupled with the knowledge to analyze data and interpret results, will enable effective detection of sickness in housing systems where animals in large groups or complex environments are difficult to monitor visually. However, it should be mentioned that producers are unlikely to treat individual hens in contemporary, large, modern systems in contrast to dairy systems or swine husbandry operations. The difference between systems most likely related to the individual value of a single laying hen *versus* that of a dairy cow or gestating sow. The authors believe development of technology that allows tracking of individual hens could make identification of problems and delivery of appropriate care a possible practical application for tracking technology. However, this review is focused on providing an understanding of how to use such technology as a research tool under these challenging conditions rather than how it could be adapted for on-farm commercial use.

Others have pursued metrics where aggregate individual measures of behavior have been used as indicators of animal health. For instance, the optic flow system [19] assesses aggregate movement of individuals within flocks to provide a single metric of motion for the entire flock; similar data could be collected in terms of vocalizations [20]. However, in these scenarios, the individual-level information is not accessible and, as it is not within the remit for the objectives of this paper, it will not be discussed further.

## 4. General Considerations when Using Bird-Mounted Sensor Technology 

The use of remote tracking technologies should require minimal intervention and minimize interference with the normal behavior of birds. However, there is a risk that the devices attached to the birds are invasive, restrictive, or pose a risk of entanglement and injury. Therefore, it is important that the systems be validated to determine whether they cause any behavioral changes and if so, to what degree. 

Studies of wild birds have shown that bird-mounted sensors can alter behavior, social interactions, mate selection, visual signal communication, body condition, and in some cases, cause physical injury, reviewed by [21]. In research involving commercial poultry, few published studies have assessed the effects of tracking devices on the behavior of the birds. Gebhardt-Henrich *et al*. [22] examined the effects of the magnetic fields generated by radio frequency identification (RFID) antennas on pop hole use by monitoring the number of entries and exits in two pop holes, one switched on and one switched off, and alternating which pop hole was on/off. The authors found no effect of the electromagnetic field on the frequency with which the birds used the pop holes. Similarly, Hartcher *et al*. [23] used video cameras to record indoor and outdoor movement before and after installing RFID equipment and found that the ranging behavior was not affected by the tracking system. In assessing the effect of tracking devices worn within backpacks (10 g total mass), Daigle *et al*. [24], found a reduction of feeder and drinker use by laying hens with an increased use of nest boxes and perches during the first 48 hours after birds were fitted with sensors. However, the authors did not find any effects on the agonistic interactions or body mass after 16 days of wearing the devices. While these studies show little to no effect on the use of different resources and the behavior of the birds, the continuing development of remote tracking technologies demands systematic validation as systems continue to evolve.

In animal ecology studies it is a common recommendation that the body-mounted equipment weighs less than 5% of the animal’s body weight to minimize effects of the tracking devices on behavior and performance [25]. The authors of the current work recommend that this threshold (*i.e.*, <5%) be adopted for studies of this nature in commercial poultry. Nonetheless, validation should still be performed to ensure gross alteration of behavior is not occurring as changes have been reported, even when equipment weighs less than the suggested threshold. For example, in sea birds (*Calonectris diomedea*) a reduction of 1.6% in body mass has been reported in birds wearing a tracking device (geolocators attached by a leg band) weighing 12 g, which represented only 1.5–2% of the birds body weight [26]. Similarly, a study in the flightless Takahe (*Porphyrio mantelli*) found that daily energy expenditure increased 7.7% when using bird-mounted radio transmitters that weighed an average 1.8% of the birds` body mass though time budgets of the birds were not affected [27]. The authors suggested that the increased energy expenditure could result from increased thermoregulation due to feather disruption by the tag and harness, a highlight of the importance to consider not only the mass of the devices but also the method of attachment. 

Dennis *et al*. [24] compared the effects of different marking methods (metal leg-bands, metal wing-band or livestock marker on the tail) on body weight, behavior, physiology and immunity of pair caged laying hens. The authors marked only one bird within a pair and found a significant reduction in body weight, increased fluctuating leg asymmetry and reduced immune function in birds marked with metal leg-bands compared to control un-marked pair housed birds [24]. Similarly, Liste *et al*. [25] found that markings that created a heterogeneous physical appeareance (marking the back of the head with a black dye in 0, 30, 50, 70, or 100% of the birds) lead to increased levels of aggression received and given in laying hens. These studies suggest that marking only a portion of the birds in a group can lead to physiological and behavioral changes in marked birds and affect how other birds perceive and interact with them. 

Due to the high costs of the remote tracking equipment and the large group sizes in which poultry are usually kept, it is common practice to select a sample of birds for assessment. Thus, beyond concerns that tracking equipment can affect bird behavior, researchers must also ensure that the sampled birds are representative of the larger group population. While a random sampling may be used, this is often confounded in that some birds may be easier to catch (e.g., slower or naturally less fearful birds) making the sample population non-random and not representative. To minimize this effect, efforts should be made to ensure the selection of birds is stratified across the pen by pre-selecting focal birds during population of the barn, e.g., every nth bird placed into a pen. Alternatively, researchers could identify areas of the pen from which they would select a proportion of focal animals [28,29,30]. Others have considered social structure in the selection process, a factor that is likely to be particularly powerful in smaller groups of birds (e.g., less than 50). Daigle *et al*. [18] selected birds based on body weight and suggested that comb characteristics should also be considered as both characteristics have been shown to relate to social status and increased probability of winning a fight [31]. 

## 5. Methodology for Identifying Behavior and Location—Technical Details and Examples

### 5.1. Behavioral Identification with Accelerometers

Information on how individual non-cage laying hens use space and the spatio-temporal variation in hens` space use is poorly understood. The dearth of information results from the methodological challenges inherent to describing individual animal behavior in group settings. By placing body-worn sensors on a representative proportion of the population and adopting analytical techniques used by other disciplines (e.g., Geographic Information Systems (GIS)), we may be able to better capture the responses of individuals housed in large groups. Modeling the spatial configuration of hen behaviors can provide insight into the general welfare of the individual. For example, feeding (consuming food from a feeder) and foraging (searching for and/or consuming food found in litter using the feet and beak) are both behaviors that hens are motivated to perform and are required for survival in natural settings [32,33]. Preening (*i.e.*, a maintenance and comfort behavior where feathers are cleaned with the beak) can be considered a comfort behavior and has been observed to be performed more often in the presence of familiar conspecifics [34]. Additionally, based on previous studies, it is clear that hens make different choices with regard to where and when they choose to perform these welfare-relevant behaviors based upon their perception of the environment [35,36]. Technology that would allow identification of these behaviors in large groups (and their associated frequency) as well as influencing factors would allow for a paradigm shift in the way we evaluate animal welfare. 

As one tool to identify behaviors remotely, accelerometers, or sensors that estimate acceleration along one or more axes, can be used to estimate velocity and displacement. Body-mounted accelerometers respond to acceleration resulting from movement of the individual as well as gravitational acceleration. When equipped appropriately, accelerometers have the potential to remotely detect performance of specific behaviors by individuals using movement data [24]. Accelerometers have played an important role in detecting specific behaviors in animals including cattle [37] and chickens [38,39] and in describing general activity levels in species such as dogs [40], chickens [41] and elephants [42,43]. 

The major challenge involved in using accelerometers to accurately detect the performance of specific behaviors in chickens is two-fold, both of which stem from the size of the subject. First, the size and weight of the sensor must not cause any significant change in the bird`s natural behavior, a topic that was discussed with greater detail in an earlier section (#4) of this manuscript. The limitation is not a problem in larger animals (e.g., cattle) and hence sensors equipped with better processing capabilities and larger batteries that increase the total size and weight can be used in these species without concern. Second, due to the relatively small size of hens and general movement patterns, the magnitude of acceleration produced by hens during movement is considerably smaller than that of larger animals and thus the state-space of accelerometer values obtained is relatively smaller. The brief and jittery movements produced by chickens result in a weak correlation between the accelerometer data and the activity of the individual and require their own unique protocols. To identify target sets of behavioral activities, specific machine learning mechanisms should be used on the features extracted from hens’ activity data.

**Figure 1:**
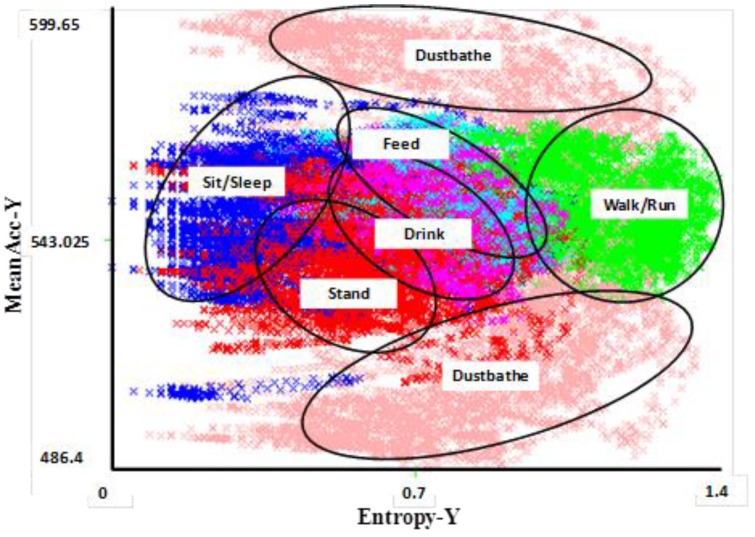
Visual clustering of activities in a 2D feature space for Y and X axis accelerometer data [38]. The different behavioral activities appear as different distinct clusters, allowing them to accurately be distinguished from each other. The most difficult behaviors to distinguish are feeding and drinking, both of which involve a stationary body with pecking motion of the head (upwards in the case of drinking and downwards in the case of feeding). Data were collected from six sensor-wearing hens over several days to capture multiple performances of an activity by each hen. The clusters shown here correspond to classifications using the 50% test dataset.

Data from accelerometers have the following attributes: time, acceleration along the x-axis, acceleration along the y-axis, and possibly acceleration along the (third) z-axis though this review focuses on work conducted using a two-axis system. Two features, entropy and mean, can be extracted from each axis within a specified time window giving a total of four attributes for use in machine learning classification in a two-axis system. These two features are important for distinguishing different activities from one another by comparing their intensities and periodicities over time (Figure 1). Generally, entropy represents the expected amount of information within a window and, the more intense the activity, the more entropy is recorded. The mean entropy is calculated as the average value within the time frame.

Banerjee *et al.* [38] sampled individual hen behavior using a body-mounted sensor at a rate of 10 Hz (*i.e.*, 100 ms sampling interval), and tested window sizes of 30 to 40 samples (*i.e.*, over 3 s and 4 s time periods, respectively, based on the sampling rate). A window was used for data analysis rather than raw accelerometer data, as windows can capture several cycles of the behavioral activity of interest within a single sample (e.g., multiple steps during walking or sequences of pecking during feeding or drinking). Overlapping windows can also improve detection accuracy. In their study, high intensity activities (e.g., walk/run) consistently reported higher entropy values than lower intensity activities (e.g., feed or drink). Similarly, there was a distinct difference in the entropy values between sitting/sleeping and feeding. The duration of individual data collection sessions varied from 30 s to 20 min depending upon the naturally occurring duration of the target activity. Data were collected until at least 15 min of data for each activity was obtained. For two behaviors that were difficult to induce in a timely manner and that occurred for very short durations, less data were obtained (dust bathing = data from 3 of 6 hens; drinking = 7 min total data). Data were labeled according to the activity being performed in a semiautomatic manner. In addition to data being collected by the sensor, a human observer recorded timing of the performance of specific activities. The acceleration data collected by the sensor between start and stop times noted by the observer for a certain activity were labeled accordingly. To avoid mislabeling of data due to human reaction time being slower than that of the sensor, data within 5 s of the start and end times of an activity were discarded. Labeled data from all hens were combined and 50% of the data was used to train the classifier. The remaining 50% of the data were used as a test data set.

In order to identify specific behaviors using entropy and acceleration, the accuracy of the software program used to classify the data as a particular behavior and the underlying criteria used to distinguish between behaviors of interest must be evaluated. In order to classify individual hen behavior, Banerjee *et al.* [38] evaluated the accuracy of multiple processing methodologies including: Decision Tree (J48), Neural Network, Radial Basis Function (RBF) Network and Naïve Bayes Tree. When behavioral classifications from these methodologies were compared with behavioral data obtained from watching video recordings of the sensor-wearing laying hens, the Neural Network approach using six hidden neurons provided the best results based on both windows of three and four seconds. This methodology was used for subsequent analyses of data generated by this sensor system.

**Figure 2:**
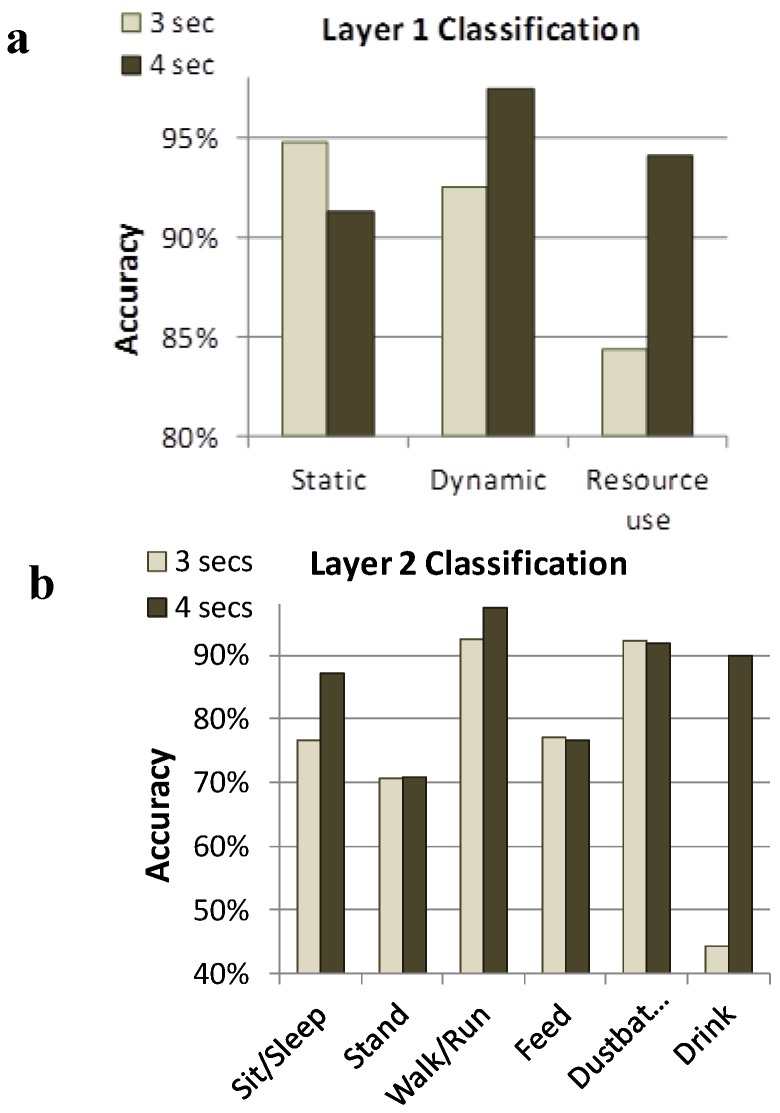
Layer 1 (**a**) and Layer 2 (**b**) classification systems to remotely identify performance of behaviors within 3 and 4 seconds windows [38]. Layer-1 accuracy was higher than Layer-2 accuracy, and in most cases, 4 s windows were more accurate than 3 s windows. Static behaviors (sit/sleep and stand) could accurately be distinguished from dynamic behaviors (walk and dust bathe) and both of these categories could be distinguished from resource use behaviors (feed and drink).

A layer-classification approach can also utilize a hierarchical approach to classify sensor output collected from hens across multiple days. Banerjee *et al.* [38] used a 2-layer approach where the top-layer (Layer 1) classification separated behaviors based on whether they were static, dynamic, or indicative of resource use (Figure 2). Within the three classifications used for Layer 1, a second layer (Layer 2) allowed discrimination of output into six separate activities: sit/sleep, stand, walk/run, feed, drink and dust bathe. In moving to Layer 2 classification, most of the misclassifications occurred within the individual classes identified in Layer 1, not between separate classes. For example, the majority of errors in detecting standing were caused by falsely classifying the behavior as sit, another behavior in the static class. Similarly, the majority of misclassifications of drinking data were as feed, another behavior in the resource use class. Using a tri-axis accelerometer to capture the z-axis would likely remedy some of these problems.

When combining the window size and classification criteria, Banerjee *et al.* [38] achieved a Layer 2 overall accuracy of 78.46% and 82.61% using the Neural Network in terms of correctly classifying the behaviors for the 3 s and 4 s windows, respectively. Therefore, accuracy increased with window size, however there is a cost that must be considered against the potential benefit. For example, if the sample window is too large, behaviors characterized by shorter durations may not be captured in isolation but the window will contain acceleration data related to fragments of preceding or following behaviors. Alternatively, if the window is too short, the sensor system may not generate enough information for accurate classification of the behavior by the software. Therefore, windows of varying length need to be tested when determining a sensor output algorithm that is appropriate for the target behavioral activities in question. In addition, there is some degree of variability in how individual hens perform even very fundamental motor patterns such as pecking and there is potential to misclassify behaviors with similar activity signatures (Figure 2). Therefore, accuracy of behavioral detection by such a sensor may require further training of the system using more data from each individual. 

### 5.2. Background, Radio Frequency Identification 

Potential applications for RFID systems are numerous and can be used in a variety of contexts of which two are examined in this review: registration of passage through a pop hole and egg-laying in a nest box. In brief, RFID systems use a uniquely coded identifying unit, typically referred to as a transponder, which when in proximity to a powered antenna, is registered by a reading unit and the transponder identity sent to a central computer. For the purpose of this review, we have focused on applications where the location of a hen is registered within a defined area (*i.e.*, nest box) or by linking multiple antennae to record movement from one defined area to another (*i.e.*, veranda/free-range). Specific RFID systems will have varying capacities, including the proximity with which the transponder can be identified by the antennae and whether multiple transponders can be identified simultaneously. In terms of simultaneous registration, a high frequency (like 13.56 MHz *versus* the low frequency 134.2 kHz) can detect several transponders simultaneously on the same antenna with the help of an additional protocol, commonly referred to as an anti-collision protocol (e.g., International Organization for Standardization (ISO) #15693). In brief, several variations exist at present. The first operates where the transponder responds in a slightly changed frequency and the reader goes through each frequency to identify them. A second variation allows the transponders to answer with a specific time lap during which the reader tries to read them one after the other. A last variation allows the reader to tell those transponders that have been read to shut down for the ongoing reading in order to read all remaining transponders. 

The RFID systems function where the transponder with its antenna is energized by the electro-magnetic field produced by the reader antennae, which then sends a uniquely coded signal to the receiving unit that decodes the transponder number alongside the time and date. In this way, the RFID systems have a major advantage over other systems in that the transponders (located on the bird) do not require an external power source and can therefore be minimized in size and weight. Assuming that the antennae are provided with uninterrupted electrical power, the system can continue collecting data for extended periods of time, e.g., the approximately 50 weeks of a laying cycle [30,44]. The RFID systems can generally be used as a mobile system, allowing for relatively easy installation at varied locations for a period of time, after which they can be disassembled, cleaned, and moved to another location. The system’s mobility is reduced in locations where the presence of water or metal can dampen the signal requiring additional setup time to ensure the transponder is read with the required accuracy. The system’s ability to transmit through these mediums, and thus the ease in setup, will vary with the transmitting frequency used. Lastly, the speed of the animal being tracked (or the attached transponder) is also a major consideration in deciding what type of RFID system should be used and their arrangement. Speed is particularly relevant for tracking passage in/out of areas (such as the pop hole) and is more thoroughly discussed below. 

### 5.3. Range Use with RFID

Multiple groups have independently used RFID technology in order to record ranging behavior and the associated occurrence of hens passing through a pop hole within a commercial setting with similar but variable approaches [30,44,45,46,47,48,49,50,51]. The effort by Gebhard-Henrich *et al*. [45] is described here in the greatest detail as the accuracy of the system was validated in an accompanying paper [22], the system registered actual passage of hens through the pop hole *vs*. presence in the pop hole alone (as with [30]), and the setup minimized changes to the pop hole itself (as with [46,47,48,49]). A second validated system, also described in detail, that did not require adjustments to the pop hole dimensions is “*the wide electronic pop hole*” [50,51]. Relative benefits and drawbacks of each system are discussed below. 

Specifically for the effort by Gebhardt-Henrich [45], a low frequency RFID system (Gantner pigeon system, Benzing, Schruns, Austria) (125 kHz) was used to study ranging behavior in 12 flocks of laying hens where the aim was to monitor individual bird usage of outdoor areas (verandas and free range areas) without altering pop hole number, dimensions, or management. Across the various flocks, between 5 and 10% of the birds were tracked. Antennae were placed on either side of each pop hole linking the house/veranda and veranda/free-range. The width of the pop holes ranged from 1.2 to 4.6 m and, depending on the size of the pop hole, up to 12 antennae were put side-by-side to cover the entire width of both sides of the pop hole. The inclusion of antennae on either side of the pop hole was necessary to determine the direction of a hen`s transition between two areas and therefore required registration of two events—both entrance into the pop hole in one area (e.g., inside the house) followed by exit from the pop hole into a second area (e.g., to the veranda). 

An alternative effort by Thurner *et al*. [50] which registered pop hole usage of all birds in the flock with a system termed the “wide electronic pop hole” used a high frequency (13.54 MHz) RFID technology setup. The system was required to be inserted into the pop hole and tuned when positioned (tuning board ISC.MAT-A, Feig Electronics, Weilburg, Germany). Compared to the system used by Gebhardt-Henrich *et al*. [45], “*the wide electronic pop hole*” uses two antennae to determine the hens` direction and is designed as a tunnel with a length of 1.0 m, a cross section of 0.35 m by 0.70 m, and a width that can be changed according to the pop hole size. The identification reliability was evaluated using video recordings over several days from the whole flock and resulted in an identification reliability of 97.6% for a width of 70 cm (n = 3,113 passages), 99.3% for a width of 55 cm (n = 606 passages) and 99.8% for a width of 40 cm (n = 582 passages) [50]. In this study, hens that had lost the transponder or had a defective transponder were included in the reliability evaluation.

While an arrangement with RFID antennae on either side of the pop holes provides a high degree of coverage to ensure accurate registration of hens, it requires a relatively high number of antennae and associated hardware that unfortunately increases the purchase costs. For instance, a typical barn with 25,000 hens may have 40 pop holes and if each of those pop holes requires 12 antennae, the cost quickly becomes prohibitive. As an alternative that allows the relative benefits of RFIDs with reduced costs, researchers have employed a methodology using antennae that were placed in the pop hole itself. Using this configuration, Richards *et al*. [27] assessed half of a single flock (approximately 6000 birds in the surveyed group) while Thurner *et al*. [50] assessed all bird in small groups between 300 and 400 hens per group. While this configuration reduces the number antennae needed, the major drawback is that the system is unable to identify the hen’s direction of motion when leaving the pop hole and thus allows only recording of the hen’s presence in the pop hole. In other words, when a hen was registered by the antennae, the authors had no way of identifying whether it originated from inside or outside the house or where it returned to that originating location. Despite this drawback, the effort by Richards *et al*. [30] and Thurner [50] had results which seemed to parallel that of Gebhardt-Henrich *et al*. [45] (e.g., all found a bimodal distribution in range use), thus the arrangement is an attractive option if needed. 

Another variation that substituted placement of antennae on either side of the pop hole was employed by Hernandez *et al*. [46] who used light beam sensors in combination with an RFID configuration to register passage. The inclusion of the light beam sensors (PD70CNT12, Carlo Gavazzi Automation S.p.A, Italy) installed inside and outside of the pop hole passageways allowed for recording direction of hen movement (*i.e.*, in or out of the barn by registering a blockage of the beam) with a single antenna in groups of 200 [46,49] and 600 (unpublished data) laying hens. During the initial setup, it became apparent that the high motivation to gain access to the range area caused multiple problems. A large number of hens rushed to go through the pop holes at the same time creating a bottleneck, resulting in several missed RFID readings that were identified by parallel video observations. Comparing video recordings and RFID output found that, when birds got stuck in front of the pop holes, some birds at the back jumped over those stuck at the front resulting in a distance beyond the antennae`s reading range. Furthermore, when birds ran (rather than walked) through the pop holes, some failed to step on the antenna with the leg containing the transponder and were not registered. These problems were addressed in the final set-up by altering increasing the height and reducing the width of the pop holes. By setting the pop hole height at 30 cm above the ground, “bird jumping” was limited and encouraged birds to hop into the pop hole and land with both feet directly on the antenna ensuring an RFID reading. In addition, reducing the width of the pop hole limited access so that only one bird at a time could pass through the pop hole. Increasing the height and reducing the width of the pop holes increased the bottleneck effect, but since bird passage through the pop holes was better controlled, the data more accurately reflected bird movement. Besides affecting registration of hens, pop hole sizes may also affect the behavior of the hens. Thurner [48] found with a system termed the “*narrow electronic pop hole”*, which allowed only passage of a single bird at a time, that hens did not use these narrow pop holes to the same extent as relatively wider pop holes. 

Other problems encountered with using RFID systems are birds that stay within reading distance of the antennas for extended periods of time, which can result in several hundred readings from the same bird during a single visit to the pop hole. The problem can be addressed with software modifications by reading RFID tags only once while a hen remains within the reading range [46] or by restricting data to one reading every minute for each bird [30]. The protocol can also be modified so that the collected data is filtered at a stage subsequent to actual transponder registration (*i.e.*, all registrations are recorded) allowing collected data to be continually re-assessed for the most appropriate filtration. A final problem is that a bird may pass through the pop hole but return (without leaving the reading range of the antenna), an event that would appear as the bird going outside several times but not coming back in, which relates to the pop hole usage problem described earlier. However, in this scenario, the use of light sensors can allow the operator to exclude consecutive OUT readings that do not have matched IN readings (or *vice versa*). Another way to solve this problem is to have two RFID antennae with high reading rates and an algorithm that can identify when the bird is entering and leaving the pop hole from the same side [48]. 

More generally, a major limitation of RFID systems used for research purposes such as these (with rapid movement of hens) is the velocity of the moving transponders over the antennae. In validation testing, accuracy of registration began to fall sharply for tags moving faster than 1.5 m/s, though adapting to a 32-bit system could increase the maximum speed to 3.2 m/s [22]. In order to put this speed into context for likely applications, the median calculated speed for tagged brown hens on one particular farm when passing the pop holes was 1.5 m/s, but some hens reached more than 4.5 m/s [22]. Thus, with a 32-bit system, most laying hen movements would be expected to register. The greater speed of hens could also decrease the likelihood of registration where wider gaits of fast-moving-hens might have moved the transponder outside the recording range of the 300 mm wide antenna, a problem mentioned earlier [46]. In the work of Gebhardt-Henrich *et al*. [22], the probability of registration was higher when hens were leaving the house or the veranda than when they returned (leaving the house: 94.3%, returning: 83.5%, overall in terms of days where outdoor access was possible; Wilcoxon test *p* < 0.0005, N = 10; from the veranda to the pasture: 94.8%, back to the veranda: 83%, Wilcoxon test *p* < 0.0005, N = 10; [22]). The speed of the hens passing the pop holes was probably higher when they returned to the veranda or the house because of frightening events or because the farmer chased them inside at the end of the day. 

Probability of registration was also influenced by other attributes of the pop holes. For instance, hens were more likely registered when pop holes were reached through ramps than when they were on the same level as the floor of the house. Possible solutions include laying two antennas side-by-side (creating, in principle, a larger antennae), fixing transponders on both legs to increase the probability of registration, or use of an RFID system with a higher frequency. Furthermore, larger transponders and antennae have larger reading ranges, and the time the transponder needs to be read will vary with the frequency and protocol. Therefore, a person should calculate the time the transponder is within the antenna field and whether this time is long enough to complete one reading cycle. 

### 5.4. Tracking Egg-Laying with RFID Systems

**Figure 3:**
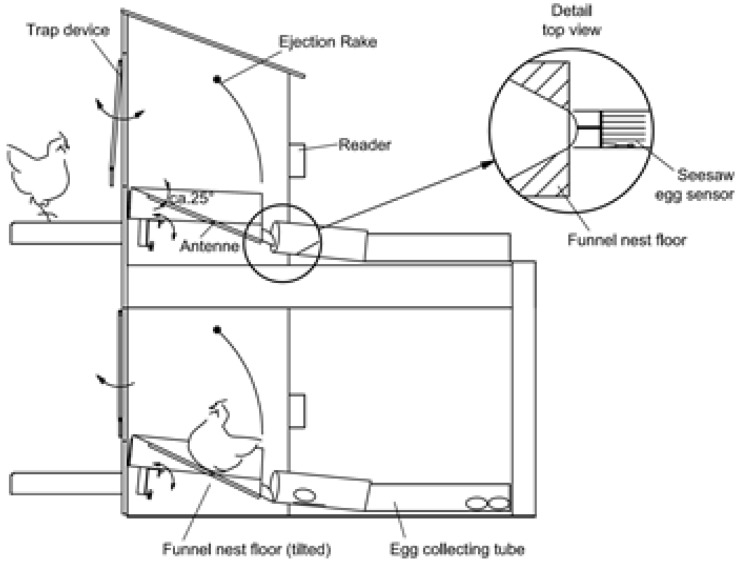
Schematic diagram of the funnel nest box [55].

Although attempts have been made to develop nests for individual productivity recording of laying hens [52,53,54], to the authors` knowledge, individual productivity within large layer flocks over extended periods has only been successfully tested with the use of trap nests or funnel nest boxes [55,56]. The funnel nest box is a single nest box that can be used in group housing systems for recording the individual laying behavior and performance as well as individual egg quality parameters since it allows an assignment of each egg to the hen (Figure 3). The funnel nest box is based on low frequency RFID technology (134.2 kHz) as described above (Section 5.2). A trapezoid shaped antenna (length 240 mm, upper width 210 mm, lower width 100 mm) that reads the 23-mm glass transponder (Texas Instruments, HDX, ISO 11784/11785) on the hens’ leg is placed under the funnel floor. Each antenna is powered by a single, synchronized RFID-module, which initiates a reading at ten times per second. Four RFID-modules together with eight input channels for sensors (seesaw egg sensor and for double nest occupations, double tilt sensor) are combined to a fourfold reader unit [57]. Up to 50 reader units can be connected via a RS485 bus-system to a central computer that sends registered data. The central computer controls the reader units, collects and analyses data with software packages specifically developed by researchers within the “Bayerische Landesanstalt für Landwirtschaft” in Germany. Though not available commercially, the software is being used for research purposes only.

**Figure 4:**
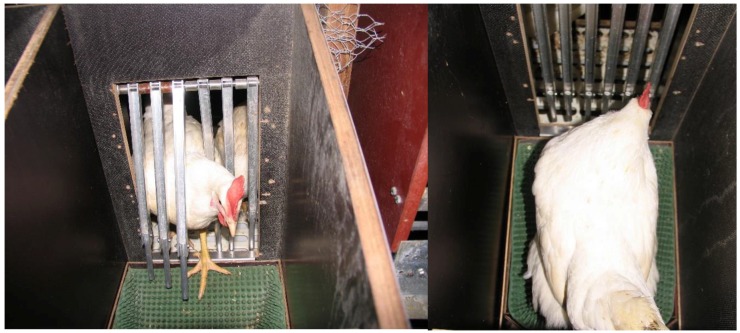
View of a hen entering the funnel nestbox (**left**) and positioning herself to lay an egg (**right**).

When the system is operational, the hen enters the nest box via a trap device that is built with an entranceway composed of six aluminum rods which are suspended above the nest box in such a way that they can swing in and out of the nest box (Figure 3). The trap device is responsible for separating the hens and for locking the nest box while occupied. After a hen has entered the nest box, the funnel floor tilts to the back, far enough to lock the trap device. Once closed, the trap can be opened (by the hen inside the nest box) by pushing it towards the outside of the nest box, a mechanism that also prevents hens from outside entering the locked nest box. A transponder, attached to the hen`s leg using a commercially available leg band (RoxanID, UK), is read by the antenna while the hen is in the nest box. The transponder signals are used to determine the number of nest box visits per day by each hen and the duration of each nest box visit. The system is capable of recognizing (by mass and sequence of transponder readings) when the nest is occupied by more than one hen. For this condition, the double tilting floor has to be adjusted to the lowest weight of a producing hen in the flock and will then register automatically if there are two or more hens in the nest box. The floor will tilt the first time when one hen is in the nest box locking the trap device. As soon as there is more than one hen in the nest box, the double tilting floor will tilt a second time (due to the greater weight) and press an attached spring sensor generating a signal that is recorded by the reader. Combining this signal and the sequence of transponder readings, up to 60% of the double nest occupations can be detected. From the undetected double nest occupations, 25% were critical, meaning that there was an egg laid. By choosing the rate of signals from the spring sensor or the minimum duration of the altering transponder readings, the researcher is able to make appropriate adjustments. The funnel-shaped nest floor is well tolerated by the hen and encourages her to assume a position with her head directed towards the nest exit causing the egg to roll from the nest immediately after laying (Figure 4). The exit of the egg ensures that the egg of the current hen is not confused with that of the next hen.

After the egg exits the nest, it is registered by a seesaw egg sensor that is integrated into the egg collecting tube (100 mm diameter, closed pipe on either side of the seesaw egg sensor) where the eggs are stored in the order of lay for each nest box until the end of the daily laying time. The seesaw egg sensor also activates an attached spring sensor that is linked to a sensor input channel of the reader unit and thus registers the oviposition time for each egg. At the end of the daily laying time, the nest boxes are locked using a paddle inside the nest box. Using this system, each egg is assigned to the respective hen by combining the data from the transponder, seesaw egg sensor, and their relative order in the collecting tube.

To validate the system, video recordings were made of the hens while entering or leaving the funnel nest box and manually evaluated. Across the number of single nest boxes and size of the group housing section (4, 24 or 48 nest boxes per section), hen to nest box ratio (5.3 to 10.5 hens per nest box), and double occupation rate (0.0 to 10.5% of all recorded nesting events within a section), the reliability of correctly recording the hen’s nest entrance and exit varied between 76.1% and 97.8% (n = 2676 nest entrances and exits). The greatest reliability was associated with a hen to nest box ratio of less than seven and in flocks with less than a 5% double occupation rate. Adapting the system to allow identification of double nest occupations resulted in a correct assignment of the egg to the actual hen that exceeded 94% for all tested flocks which improved upon other efforts [56]. 

It is possible that the system described [55] may have influenced nesting behavior. For instance, laying hens using the funnel nest box appeared to stay longer in the nest box for each nest visit and show a lower number of nest box visits per day compared to a group nest box which is common in commercial housing systems (e.g., [58]). Furthermore, the number of floor eggs can be on a higher but still acceptable level (*i.e.*, up to 5% but in extreme cases up to 10%) when using the funnel nest box compared to other designs [56].

### 5.5. Radio Signal Strength (RSS)

**Figure 5:**
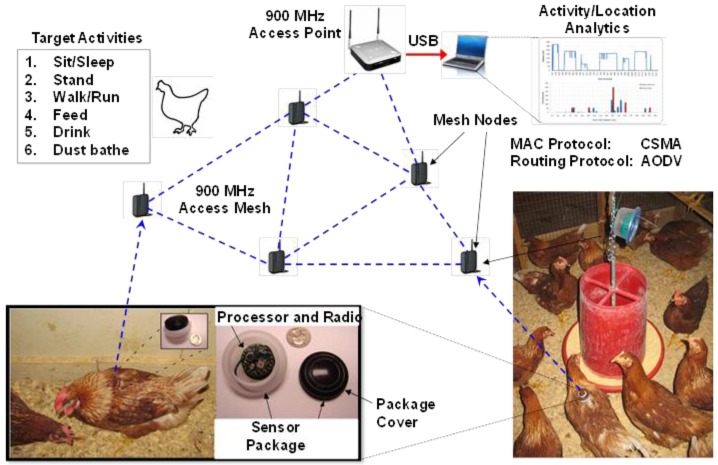
Arrangement of the various components within the body-worn RSSI sensor system. Hens wear small (10 g) sensors on their backs attached via a figure eight harness. These sensors actively transmit information to stationary receivers, which communicate with a base station. Activity and location analyses are then performed using analytical software to classify accelerometer data into specific behavioral activities and locations relative to other hens and the stationary receivers.

Radio signal strength technology employs active transmission of radio frequency sensors equipped with radio signal strength indicators (RSSI) [41]. In general, the system operates by devices (mounted on the backs of birds with a special harness) emitting a signal that is sensed by receiving units that register the signal strength which can then be used to estimate a hen`s relative location. The system discussed her used a 900 MHz radio frequency channel and a sensor card in a body-mountable package within a network arrangement (Figure 5). The total weight of the sensor package was approximately 10 g and mounted on a hen’s back within a molded casing using a figure-eight nylon harness. The casing was colored to match hen feather color and painted with a unique number for easy visual identification. After experimenting with a number of other attachments, it was found that mounting the sensor on the back of the hen resulted in maximum sensor stability while maintaining sufficient 900 MHz radio signal quality and avoiding any tissue damage to the hen or changes in behavior [24]. After attaching the sensor housing on the back, hens were allowed to acclimatize to wearing it for 48 h prior to beginning experiments. The sensor nodes ran on a 560 mAh button cell, which was able to support the system for over 50 hours on a fresh battery when used at an output radio transmission power of 5 dBm and sampling rate of 10 Hz (*i.e.*, a 100 ms sampling interval). 

Using this technology, associated data analysis has been developed to track the location of non-cage laying hens [38,41]. This particular technology has also been used in conjunction with accelerometers to identify behaviors remotely, an application discussed in Section 5.1. For the location detection as described by Quwaider *et al*. [41], the system was constantly transferring information to a base station and connected computer and thus no data was stored on the sensors worn by the hens. The constant stream of data was useful for providing instantaneous information about the hen’s location, but may not be practical in commercial implementation or where locations over large areas are monitored. Within this configuration, the area of detection for the bird-worn sensors with respect to stationary receivers was calibrated at one meter, though the stationary elements of the system are capable of a much larger range. The range was restricted to limit packet loss (*i.e.*, sets of data sent together from the mobile sensor to the base station) and missing data caused by interference of metal in the environment which radio signals are unable to penetrate. However, since detection fields radiate in a circular fashion from the center, stationary receivers sometimes had to be placed in such a way that areas of detection overlapped to avoid leaving areas where no detection was possible. This overlap created some confusion when analyzing locational output, as a hen could sometimes be detected in two locations simultaneously. In order to minimize this problem and ensure accurate signal transfer and detection, the antennae were suspended from the ceiling using PVC piping so they were one meter above the hens in a stable orientation with the antennae facing down towards the hens. 

An additional problem with this body-mounted system was that the sensors worn by the birds were active transponders and therefore required a power source. Due to the small size of the hen, the sensor and power source must be small and lightweight, such as a watch battery, which provides limited usefulness. The longest data collection sessions were 52 hours, and, to achieve this duration, the sensor was programmed to collect data at a reduced frequency during the times when the hen was known to be less active (e.g., during the nighttime when the lights were off). In contrast, the transponders of the RFID system do not require a power source but provide a restrictive detection range that is limited to the near field of the antennae where the transponder is powered and read. 

Several logistical challenges were also related to actually attaching the bird-worn sensor. The same hen would have to be identified and caught without a large flock disturbance each time the sensor system was deployed, and multiple sensor-wearing hens within a flock were needed to obtain a representative sample size. Placing the sensor on the hen’s head was an ideal location for technical success, however, behaviorally and practically, this was not feasible. The second location tested for sensor placement was around the wing, however, this orientation of the sensor caused the sensor to give a very strong signal when the hen was facing in one direction, and a weak signal when facing the opposite direction. In addition, the sensor would occasionally flip under the wing inhibiting the ability of the sensor to transmit information. Placing the sensor on the back of the hen was the most pragmatic solution, as this location provided maximum stability and signal quality without impacting the behavior or social interactions of the hen [24]. However, care had to be taken that the sensor was oriented properly within the sensor casing to ensure good signal quality and accurate interpretation of the 3D space.

## 6. Conclusions

Animal welfare is at a critical juncture with respect to how researchers and other interested stakeholders can study and assess animals within our care. Major technological and statistical innovations within the last 20 years allow us to shift our focus past that of the flock to that of the individual. With this shift, we can now examine relationships, risk factors, and causes/effects that previously would be impossible to analyze in groups that number in the thousands. In the past, our efforts were limited to individual observations of small groups of birds that would then be extrapolated to large commercial flocks. We now know the dynamics within these two frames of references are not the same, and hence extrapolation can often fail. The authors believe that, given the concerns of using small groups to identify relevant mechanisms within large groups, animal welfare researchers should challenge themselves and their colleagues to exploit the latest and most advanced technology to ensure that improved animal welfare reaches the broadest number of animals in the flock. The current paper seeks to summarize the experiences of the authors in their efforts at monitoring individual behavior so that readers can benefit from this experience and use these methodologies to achieve the ultimate goal of delivering the highest possible level of animal welfare.
